# QTL Analysis and Nested Association Mapping for Adult Plant Resistance to Powdery Mildew in Two Bread Wheat Populations

**DOI:** 10.3389/fpls.2017.01212

**Published:** 2017-07-27

**Authors:** Yan Ren, Weixiu Hou, Caixia Lan, Bhoja R. Basnet, Ravi P. Singh, Wei Zhu, Xiyong Cheng, Dangqun Cui, Feng Chen

**Affiliations:** ^1^Collaborative Innovation Center of Henan Grain Crops/National Key Laboratory of Wheat and Maize Crop Science/Agronomy College, Henan Agricultural University Zhengzhou, China; ^2^International Maize and Wheat Improvement Center (CIMMYT) Mexico, Mexico; ^3^Shangqiu Academy of Agricultural and Forestry Sciences Shangqiu, China

**Keywords:** *Triticum aestivum* L., powdery mildew, molecular marker, QTL, JICIM

## Abstract

CIMMYT wheat (*Triticum aestivum* L.) lines Francolin#1 and Quaiu#3 displayed effective and stable adult plant resistance (APR) to Chinese *Blumeria graminis* f. sp. *tritici* isolates in the field. To elucidate their genetic basis of resistance, two recombinant inbred line (RIL) populations of their crosses with Avocet, the susceptible parent, were phenotyped in Zhengzhou and Shangqiu in the 2014–2015 and 2015–2016 cropping seasons. These populations were also genotyped with SSR (simple sequence repeat markers) and DArT (diversity arrays technology) markers. Two common significant quantitative trait loci (QTL) on wheat chromosomes 1BL and 4BL were detected in both populations by joint and individual inclusive composite interval mapping, explaining 20.3–28.7% and 9.6–15.9% of the phenotypic variance in Avocet × Francolin#1 and 4.8–11.5% and 10.8–18.9% in Avocet × Quaiu#3, respectively. Additional QTL were mapped on chromosomes 1DL and 5BL in Avocet × Francolin#1 and on 2DL and 6BS in Avocet × Quaiu#3. Among these, *QPm.heau-1DL* is probably a novel APR gene contributing 6.1–8.5% of total phenotypic variance. The QTL on 1BL corresponds to the pleiotropic multi-pathogen resistance gene *Yr29/Lr46*/*Pm39*, whereas the QTL on 2DL maps to a similar region where stripe rust resistance gene *Yr54* is located. The QTL identified can potentially be used for the improvement of powdery mildew and rust resistance in wheat breeding.

## Introduction

Powdery mildew (PM), caused by *Blumeria graminis* f. sp. *tritici* (*Bgt*), is a significant disease of common wheat worldwide, reported to have caused yield losses of up to 34% (Alam et al., 2011). Since the 1980s, PM has been widespread across large areas of China, and is currently a major disease in the Northern, Yellow and Huai wheat growing regions. On average, 7.0 million ha of wheat planting areas were affected annually by PM in China from 2010 to 2015 (National Agro-technical Extension and Service Center (NAESC), 2010, 2011, 2012, 2013, 2014, 2015). Growing resistant cultivars is the most cost effective and environmentally safe approach to manage the disease (Gurung et al., 2009).

Resistance to PM in wheat is of two main types, viz. race specific and race non-specific. The former is usually effective against certain *Bgt* isolates, but ineffective to others. These types of genes usually exhibit resistance through the whole growth cycle of wheat and cause various degrees of hypersensitive foliar reactions in a gene-for-gene interaction (Flor, 1942; Hsam and Zeller, 2002). Race-specific resistance genes were widely applied in Chinese wheat breeding because of their high level of effectiveness through all growth stages of the crop. However, these genes are known to break down readily due to the selection of pathogen isolates with matching virulence genes. In contrast, race non-specific resistance is often quantitatively inherited. It usually shows resistance at the adult plant stage, hence, it is also called adult plant resistance (APR), and is more durable than the race specific resistance. So far, 82 PM resistance genes have been formally cataloged on 54 loci (McIntosh et al., 2017), but most of them are race specific and are easily overcome by new *Bgt* isolates (Leath and Murphy, 1985; Limpert et al., 1987; Li et al., 2014). Consequently, breeders have paid more attention to the development of wheat cultivars with APR to PM. So far, only three wheat PM resistance genes, *Pm38, Pm39*, and *Pm46*, are known to be race non-specific and confer partial resistance. These three genes also show pleiotropic effects on resistance to stripe rust, leaf rust, and stem rust (William et al., 2003; Spielmeyer et al., 2005; Herrera-Foessel et al., 2011). Moreover, *Pm38* and *Pm46* have been cloned (Krattinger et al., 2009; Moore et al., 2015). The multi-pathogen resistances conferred by *Pm38* and *Pm46* encode a putative ABC transporter and a hexose transporter (Krattinger et al., 2009; Risk et al., 2012; Moore et al., 2015), respectively. These pleiotropic APR genes are valuable in wheat breeding for their broad effectiveness and potential durability.

In addition, over 120 PM resistance quantitative trait loci (QTL) have been identified using molecular markers in wheat (Li et al., 2014; Hao et al., 2015). However, some of these QTL might be the same due to their chromosomal location at approximate vicinity. Marone et al. (2013) developed a high-density integrated map for the projection of PM resistance genes, and identified 24 MQTLs containing 2–6 initial QTLs on 15 chromosomes through a QTL meta-analysis. The chromosomes 2B and 7A showed abundant PM resistance loci. Li et al. (2014) constructed an integrated linkage map using mapped QTL with PM and leaf rust resistance based on wheat consensus maps, and found eight resistance QTL clusters on chromosomes 1A (2 QTL clusters), 2A (2 QTL clusters), 3A, 4A, 5A, and 5B, respectively. Overall the A genome contained more PM resistance QTL than the B and D genomes.

Francolin#1 and Quaiu#3 are high yielding spring wheat lines developed by the International Maize and Wheat Improvement Center (CIMMYT), and have been widely used as parents in the CIMMYT wheat breeding program. They do not carry effective seedling resistance genes to PM as they displayed high infection type (IT) against Chinese *Bgt* isolates E09 and E20 at seedling stage (IT = 3 based on the 0–4 scale). However, the two lines exhibited stable resistance to those isolates at the adult plant stage, indicating the presence of typical APR to PM. QTL analysis for APR to stripe rust and leaf rust in Francolin#1 and Quaiu#3 were previously analyzed by Lan et al. (2014) and Basnet et al. (2014), respectively. However, the genetic basis of resistance to PM in the two parents remains unknown. The objectives of our study were to: (1) investigate the genetic basis of PM resistance in Avocet × Francolin#1 and Avocet × Quaiu#3, and (2) identify and characterize the major APR loci for PM resistance in these populations through joint and individual inclusive composite interval mapping approach.

## Materials and methods

### Plant materials

In the present study, 196 and 195 F_5_ recombinant inbred lines (RILs) from Avocet-*YrA* × Francolin#1 and Avocet-*YrA* × Quaiu#3 crosses, respectively, were used to understand the genetic basis of resistance to powdery mildew. Francolin#1 (Waxwing^*^2/Vivitsi) and Quaiu#3 (Babax/Lr42//Babax^*^2/3/Vivitsi) displayed moderate levels of stable resistance to powdery mildew at the adult plant stage. Whereas, Avocet-*YrA* (hereafter termed Avocet for simplicity), an Australian reselected line that lacks the resistance gene *YrA*, was susceptible to currently prevalent Chinese *Bgt* isolates E09 and E20 at all growth stages.

### Greenhouse test

Seedling tests of Francolin#1, Quaiu#3, Avocet and all RILs were conducted in greenhouse using the prevalent Chinese isolates E09 and E20 in 2014 and 2016. About 15 seeds of each entry were sown in 10 × 10 × 10 cm plastic pots where three seeds of susceptible cultivar Jingshuang 16 were also included as controls. Seedlings were uniformly dust inoculated with fresh conidiophores on two-leaf stage. Infection types (IT) were scored 7–10 d after inoculation based on the improved 0–4 scale standard following Wang et al. (2005).

### Field trials

Two RIL populations were hand-sown (~70 plants per line) in a 1.5 m long single row at Zhengzhou and Shangqiu in Henan province, China during the 2014–2015 and 2015–2016 cropping seasons. Every 12th row was planted with the susceptible variety Jingshuang 16 as controls and spreader rows. Inoculations on spreader rows were carried out using two prevalent Chinese isolates E09 and E20 at the early jointing stage at Zhengzhou and Shangqiu. Disease severity was evaluated at the filling stage when the susceptible check (Jingshuang 16) reached 80% severity and the second scoring was done 4–5 days after the first evaluation. Maximum disease severity (MDS) on RILs was used in all statistical analyses and QTL mapping. The field data from the 2014–2015 cropping season at Shangqiu was not used due to poor PM development.

### Genetic and statistical analyses

To preliminarily estimate the number of PM resistance genes in two RIL populations using Mendelian segregation analysis, the RILs in each population were classified into two phenotypic categories following Lillemo et al. (2006); resistant (R), and susceptible- intermediate (SI). A line was considered R when it had a similar disease score as the resistant parent ± one standard deviation; otherwise, the line was classified into SI. The goodness-of-fit to the expected segregation ratios for two, three, four and five independent genes were tested using Chi squared (χ^2^) analysis. Moreover, the minimum number of PM resistance genes was also determined using the Wright's method (1968): *n* = *D*^2^/4.27$\sigma_{g}^{2}$, where *D* = the phenotype range of the F_5_lines multiplied by the narrow sense heritability. Narrow sense heritability of PM was calculated using the following formula: *h*^2^ = $\sigma_{g}^{2}$/($\sigma_{g}^{2}$ + $\sigma_{e}^{2}$), where $\sigma_{g}^{2}$ = *(*$\sigma_{L}^{2}$ –$\sigma_{e}^{2}$*)/r*; in this formula, $\sigma_{g}^{2}=$ genetic variance, $\sigma_{L}^{2}=$ variance of the F_5_ lines, $\sigma_{e}^{2}=$ error variance, and *r* = number of replicates.

### Map construction and QTL mapping

In total, 520 and 450 simple sequence repeat (SSR) markers were screened on the parents of Avocet × Francolin#1 and Avocet × Quaiu#3, respectively. Polymorphic SSR markers were then used to genotype the entire populations. In addition, 141 lines of Avocet × Francolin#1 and 181 lines of Avocet × Quaiu#3 were genotyped with 518 and 361 diversity array technology (DArT) markers, respectively.

Genetic linkage groups were constructed with the statistical software QTL IciMapping V4.1 (http://www.isbreeding.net/). A logarithm of odds (LOD) score of 5.0 was set to make the linkage groups. To finalize the linkage map, marker ordering and ripping were performed using RECORD and COUNT algorithms, respectively. Inclusive composite interval mapping (ICIM) was used to detect the QTL based on MDS in each environment and the means of MDS (MMDS) over all environments. LOD scores for declaring significant QTL were calculated from 1,000 permutations at the *P* = 0.05 level. A walk speed of 1.0 cM was chosen for all QTL detection. After ICIM analysis, a consensus map was constructed with QTL IciMapping V4.1 using the “CMP” tool. “nnTwoOpt” and “SAD” were used to measure linkage algorithm and criteria. Subsequently, a joint inclusive composite interval-mapping (JICIM) program (Li et al., 2011) with a stepwise regression probability of 0.001 was employed for QTL detection. The LOD threshold was calculated by 1,000 permutations at the *P* = 0.05 level. In both cases, QTL effects were estimated as the phenotypic variance explained (PVE) and additive effects explained by the QTL.

## Results

### PM evaluation in the field

Francolin#1 and Quaiu#3 were susceptible to Chinese *Bgt* isolates E09 and E20 at the seedling stage, but showed resistance at the adult plant stage. The RILs from both populations were also susceptible in seedling stage. Final MDS of Francolin#1 and Quaiu#3 were 9.8–22.7 and 26.8–31.7% across three environments, respectively, whereas it ranged from 48.3 to 78.3% for the susceptible parent Avocet over three environments. The frequency distributions of PM severity for RILs exhibited continuous variation in both populations and transgressive segregations for both increased resistance and susceptibility (Figure S1), indicating polygenic inheritance of APR to PM in both parents. Genetic analyses by Mendelian segregation analysis (Table S2) and Wright's method (Table S3) indicated the presence of 3–4 and 2–3 APR genes conferring resistance to powdery mildew in Avocet × Francolin#1 and Avocet × Quaiu#3 populations, respectively.

Pearson's correlation coefficients (*r*) for PM severities for Avocet × Francolin#1 RILs was between 0.70 and 0.73 across three environments. Highly significant correlation (*r* = 0.63–0.85) for MDS were also observed in the Avocet × Quaiu#3 population across environments. ANOVA for both populations also showed significant variance (*P* < 0.0001) among lines, environments and line × environment interactions (Table S1).

### Linkage maps construction

A total of 647 polymorphic SSR and DArT markers were used to construct the genetic linkage map in Avocet × Francolin#1 population, spanning 779 cM, 1,442 and 450 cM in the sub-genomes A, B, and D, respectively (Table S4). Twenty-four linkage groups were developed, covering all chromosomes of wheat. Meanwhile, 463 polymorphic SSR and DArT markers were used for construction of genetic linkage map in Avocet × Quaiu#3 population, covering a total of 2628.2 cM in all linkage groups (Table S4). Twenty-six linkage groups were developed, representing all chromosomes.

### QTL analysis in avocet × Francolin#1 population

**Figure 1:**
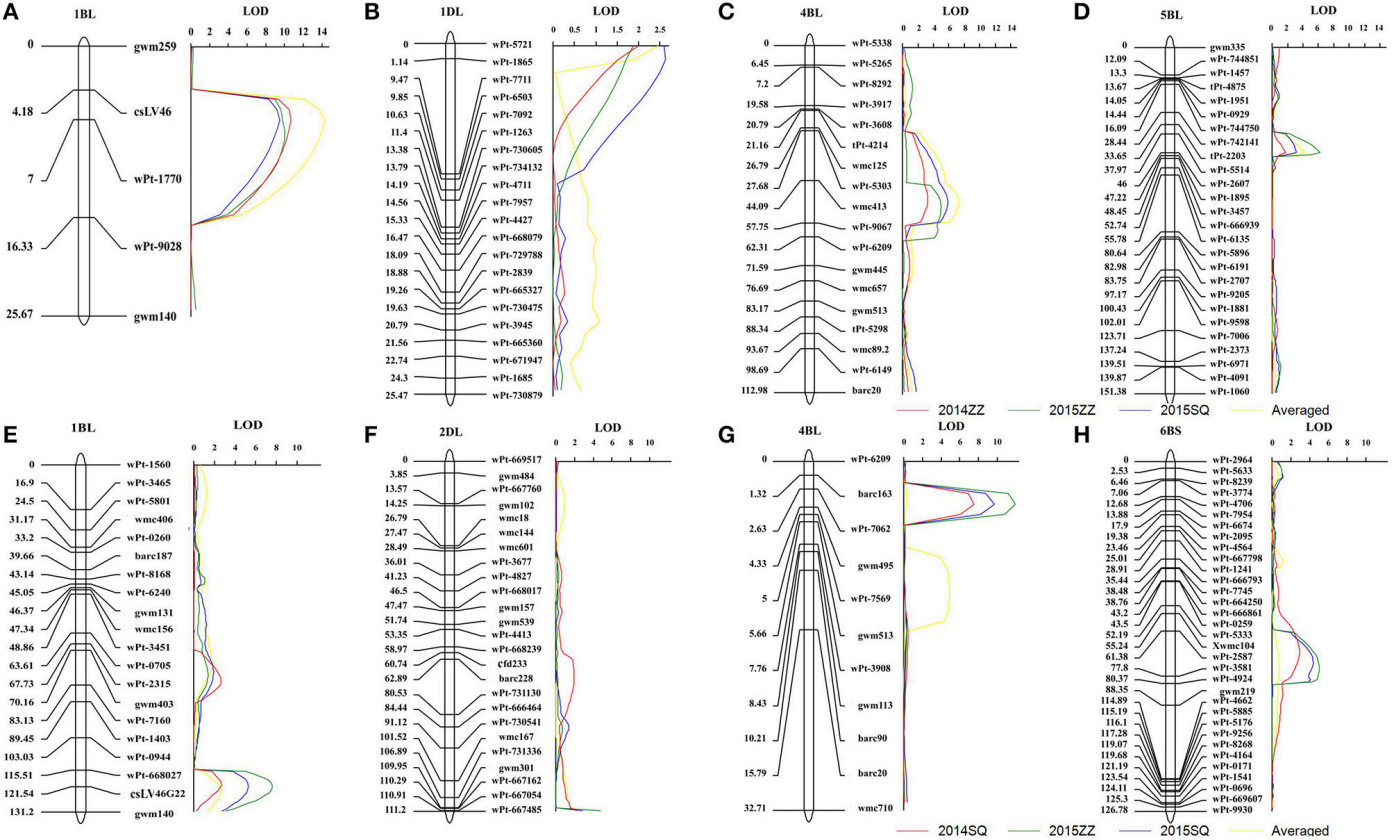
LOD contours for quantitative trait loci (QTL) to powdery mildew on chromosomes 1BL, 1DL, 4BL, and 5BL identified by inclusive composite interval mapping (ICIM) in the Avocet × Francolin#1 population **(A–D)**, and on chromosomes 1BL, 2DL, 4BL, and 6BS in the Avocet × Quaiu#3 population **(E–H)**. The significant LOD thresholds were based on 1,000 permutations. Positions (in cM) of molecular markers along chromosomes are shown on the vertical axes using cumulated genetic distances.

Four QTL for PM resistance were detected in the Avocet × Francolin#1 population (Table 1, Figure 1). The resistance genes mapped on chromosomes 1BL, 1DL, and 5BL were derived from wheat line Francolin#1, whereas the one on chromosome 4BL was from Avocet. The large-effect QTL *QPm.heau-1BL*, flanked by molecular markers *wPt-1770* and *wPt-9028*, was detected in all three environments. It explained 26.1, 20.3, 21.2, and 28.7% of phenotypic variance in Zhengzhou 2014, Zhengzhou 2015, Shangqiu 2015, and MMDS, respectively (Table 1, Figure 1A). The second consistently detected QTL with larger effect, *QPm.heau-4BL*, was located on chromosome 4BL between *wmc413* and *wPt-9067*, explaining 9.6, 10.7, 15.1, and 15.9% of phenotypic variance in the three environments and MMDS, respectively (Table 1, Figure 1C). The third QTL *QPm.heau-5BL*, located between DArT markers *wPt-2607* and *wPt-1895* on 5BL, explained 10.9, 6.2, and 7.0% of the phenotypic variance in two environments and MMDS, respectively (Table 1, Figure 1D). The fourth QTL, *QPm.heau-1DL*, mapped in the marker interval of *wPt-5721*-*wPt-1865*, was also detected in all three environments and for MMDS, explaining 6.8, 6.1, 8.5, and 8.3% of the phenotypic variance, respectively (Table 1, Figure 1B).

**Table 1:** Quantitative trait loci (QTL) for adult-plant resistance to powdery mildew detected in Avocet × Francolin#1 RIL population through bi-parental analysis.

**QTL**	**Environment^a^**	**Position^b^**	**Marker Interval**	**LOD^c^**	**PVE (%)^d^**	**Add^e^**
*QPm.heau-1BL*	2014ZZ	7	wPt-1770-wPt-9028	10.7	26.1	12.2
	2015ZZ	8	wPt-1770-wPt-9028	10.0	20.3	9.4
	2015SQ	7	wPt-1770-wPt-9028	9.5	21.2	8.5
	Mean	7	wPt-1770-wPt-9028	14.3	28.7	10.2
*QPm.heau-1DL*	2014ZZ	0	wPt-5721-wPt-1865	2.0	6.8	6.9
	2015ZZ	0	wPt-5721-wPt-1865	1.9	6.1	5.3
	2015SQ	1	wPt-5721-wPt-1865	2.6	8.5	5.9
	Mean	0	wPt-5721-wPt-1865	2.5	8.3	6
*QPm.heau-4BL*	2014ZZ	49	wmc413-wPt-9067	3.3	9.6	−7.4
	2015ZZ	52	wmc413-wPt-9067	5	10.7	−6.8
	2015SQ	50	wmc413-wPt-9067	5.8	15.1	−7.2
	Mean	50	wmc413-wPt-9067	7.3	15.9	−7.7
*QPm.heau-5BL*	2015ZZ	46	wPt-2607-wPt-1895	6.3	10.9	7.1
	2015SQ	46	wPt-2607-wPt-1895	3.1	6.2	4.8
	Mean	46	wPt-2607-wPt-1895	4.2	7.0	5.2

a *Individual (2014ZZ, 2015ZZ and 2015SQ) and mean powdery mildew severity; ZZ, Zhengzhou; SQ, Shangqiu*.

b *Map position of QTL in cM from the top of the short arm*.

c *Logarithm of odds (LOD) score for QTL peak based on 1,000 permutations*.

d *Percent of phenotypic variance explained by the QTL*.

e *Additive effects of the QTL, “+positive” and “−negative” effects suggest the resistance contributing alleles are derived from Francolin#1 and Avocet, respectively*.

### QTL analysis in avocet × Quaiu#3 population

Four QTL for APR to PM were found in the Avocet × Quaiu#3 population (Table 2, Figure 1). The resistance genes on 1BL and 4BL were mapped at proximately chromosomal positions to those identified in the Avocet × Francolin#1 population. The largest effect resistance QTL was located on chromosome 4BL, flanked by SSR markers *wPt-7062* and *gwm495*, explaining from 10.8 to 18.9% of phenotypic variance across three environments as well as MMDS (Table 2, Figure 1G). The second QTL *QPm.heau-1BL* in the marker interval *wPt-668027*-*csLV46G22* explained 4.8, 11.5, 8.3, and 6% of phenotypic variance in Shangqiu 2014, Zhengzhou 2015, and Shangqiu 2015 and MMDS, respectively (Table 2, Figure 1E). The third QTL, *QPm.heau-6BS* located in the interval *wPt-2587*-*wPt-3581* on chromosome 6BS, explained from 7.2 to 9.4% of the phenotypic variances in two environments and MMDS (Table 2, Figure 1H). The fourth QTL, *QPm.heau-2DL*, was between *wPt-667054* and *wPt-667485*, explaining 6.7 and 4.2% of the phenotypic variance in Zhengzhou 2015 and Shangqiu 2015, respectively (Table 2, Figure 1F).

**Table 2:** Quantitative trait loci (QTL) for adult-plant resistance to powdery mildew detected in Avocet × Quaiu#3 RIL population through bi-parental analysis.

**QTL**	**Environment^a^**	**Position^b^**	**Marker Interval**	**LOD^c^**	**PVE (%)^d^**	**Add^e^**
*QPm.heau-1BL*	2014SQ	121	wPt-668027-csLV46G22	2.7	4.8	4.3
	2015ZZ	122	csLV46G22-gwm140	7.6	11.5	8.2
	2015SQ	121	wPt-668027-csLV46G22	5.3	7.4	8.3
	Mean	123	csLV46G22-gwm140	2.7	6.0	6.8
*QPm.heau-2DL*	2015ZZ	111	wPt-667054-wPt-667485	4.8	6.7	6.7
	2015SQ	111	wPt-667054-wPt-667485	2.9	4.2	5.6
*QPm.heau-4BL*	2014SQ	4	wPt-7062-gwm495	7.5	14.4	−7.5
	2015ZZ	4	wPt-7062-gwm495	11.9	18.9	−10.5
	2015SQ	4	wPt-7062-gwm495	9.7	16	−10.3
	Mean	12	barc90-barc20	4.9	10.8	−9.1
*QPm.heau-6BS*	2014SQ	69	wPt-2587-wPt-3581	3	5.4	7.2
	2015ZZ	75	wPt-2587-wPt-3581	5	7.0	8.4
	2015SQ	73	wPt-2587-wPt-3581	4.4	7.9	9.4

a *Individual (2014ZZ, 2015ZZ and 2015SQ) and mean powdery mildew severity; ZZ, Zhengzhou; SQ, Shangqiu*.

b *Map position of QTL in cM from the top of the short arm*.

c *Logarithm of odds (LOD) score for QTL peak based on 1,000 permutations*.

d *Percent of phenotypic variance explained by the QTL*.

e *Additive effects of the QTL, “+positive” and “−negative” effects suggest the resistance contributing alleles are derived from Quaiu#3 and Avocet, respectively*.

### QTL for PM resistance with JICIM

**Figure 2:**
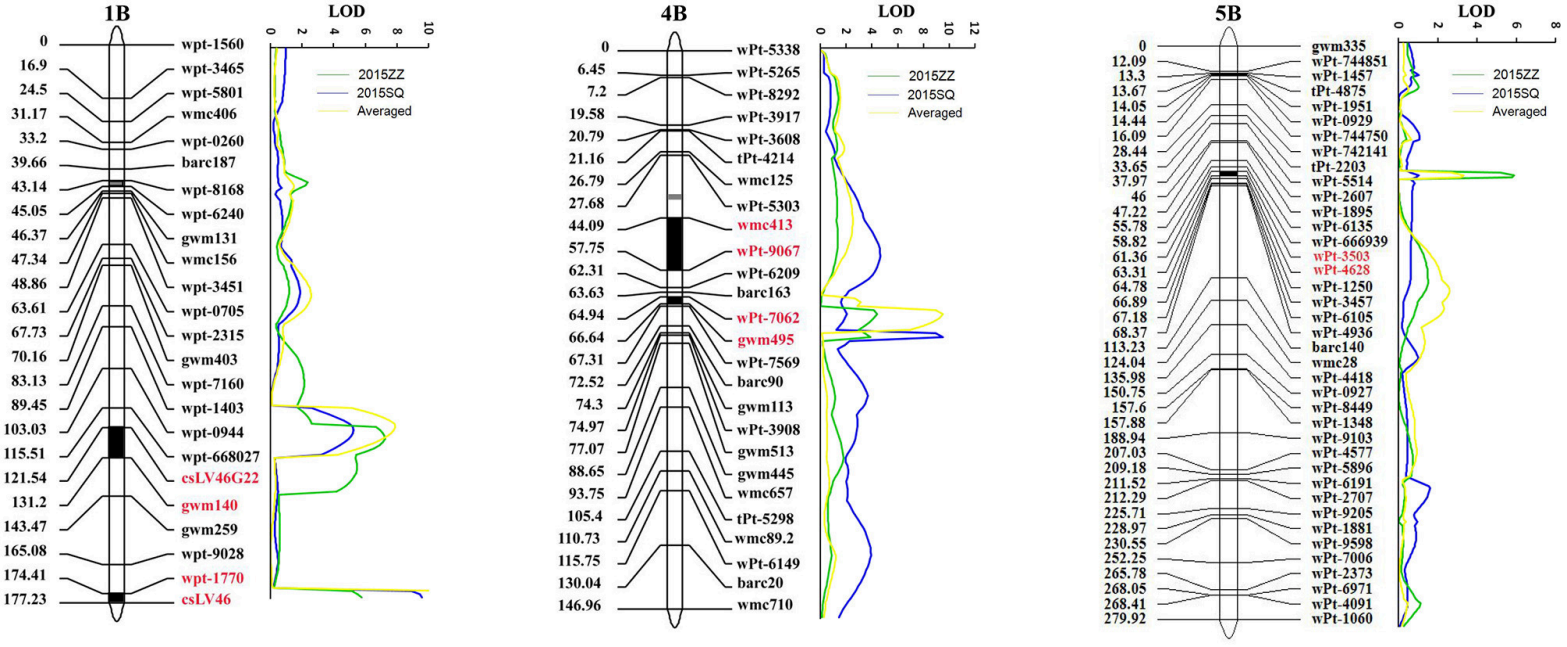
LOD contours for QTL to powdery mildew on chromosomes 1B, 4B, and 5B in the wheat nested association mapping (NAM) population by Joint inclusive composite interval mapping (JICIM).

Joint inclusive composite interval mapping (JICIM) detected five consistent QTL on chromosomes 1BL, 2DL, 4BL, 5BL, and 6BS in two environments as well as MMDS in the two populations (Table 3, Figure 2). The resistance loci on chromosomes 1BL, 4BL, and 5BL were common in both populations, whereas QTL on chromosomes 2DL and 6BS were only detected in the Avocet × Quaiu#3 population. The QTL mapped on the long arm of chromosome 4B was from the susceptible parent Avocet, explaining from 3.9 to 13.1% of phenotypic variances. The second QTL was positioned on 1BL, around the near-diagnostic marker *csLV46* and *csLV46G22*, which explained 5.2–12.9% of phenotypic variances. A third QTL with significant effects in both populations was located on 5BL between DArT markers *wPt-4628* and *wPt-3503*. The QTL on chromosomes 2DL and 6BS were only detected in the Avocet × Quaiu#3 population, explaining 4.4–8.7% and 4.3–6%, respectively, of the phenotypic variances in two environments.

**Table 3:** Quantitative trait loci (QTL) for adult-plant resistance to powdery mildew detected by joint inclusive composite interval mapping (JICIM) in the two nested association mapping (NAM) populations (Avocet × Francolin#1 and Avocet × Quaiu#3).

**QTL**	**Environment^a^**	**Position^b^**	**Marker interval**	**PVE (%)^c^**	**LOD^d^ in each family**		**Adde in each family**	
					AVS^*^Franlion#1	AVS^*^Quaiu#3	AVS^*^Franlion#1	AVS^*^Quaiu#3
*QPm.heau-1BL*	2015ZZ	177	wPt-1770-csLV46	6.8	5.6	−	−6.6	−
	2015SQ	177	wPt-1770-csLV46	8.0	9.5	−	−8.9	−
	Mean	175	wPt-1770-csLV46	15.1	11.9	−	−9.7	−
	2015ZZ	126	csLV46G22-gwm140	11.0	−	6.7	−	−7.9
	2015SQ	123	csLV46G22-gwm140	6.0	−	5.2	−	−7.6
	Mean	122	csLV46G22-gwm140	9.0	−	7.9	−	−6.9
*QPm.heau-2DL*	2015ZZ	170	wPt-667054-wPt-667485	9.7	−	5.2	−	−6.8
	2015SQ	170	wPt-667054-wPt-667485	4.4	−	3.7	−	−6.5
	Mean	170	wPt-667054-wPt-667485	5.7	−	5.0	−	−5.7
*QPm.heau-4BL*	2015ZZ	68	wPt-7569-barc90	4.4	2.0	2.4	3.9	4.4
	2015SQ	53	wmc413-wPt-9067	4.7	4.3	−	6.1	−
	Mean	68	wPt-7569-barc90	7.4	3.5	6.0	5.0	6.1
	2015SQ	74	barc90-gwm113	7.9	1.8	7.7	3.6	9.1
	Mean	68	wPt-7569-barc90	7.4	3.5	6.0	5.0	6.1
*QPm.heau-5BL*	2015ZZ	63	wPt-3503-wPt-4628	5.4	3.4	2.4	−5.1	−4.4
*QPm.heau-6BS*	2015ZZ	63	wPt-3581-wPt-9881	6.7	−	4.5	−	−5.9
	2015SQ	72	wPt-9881-wPt-9231	4.4	−	2.9	−	−6.6
	Mean	63	wPt-3581-wPt-9881	5.0	−	5.2	−	−5.5

a *Individual (2014ZZ, 2015ZZ, and 2015SQ) and mean powdery mildew severity; ZZ, Zhengzhou; SQ, Shangqiu*.

b *Map position of QTL in cM from the top of the short arm*.

c *Logarithm of odds (LOD) score for QTL peak based on 1,000 permutations*.

d *Percent of phenotypic variance explained by the QTL*.

*^e^ Additive effects of the QTL, “+positive” and “-negative” effects suggest the resistance contributing alleles are derived from Francolin#1/Quaiu#3 and Avocet, respectively*.

### Effect of *QPm.heau-1BL* and *QPm.heau-4BL* in two populations

Based on the closely linked flanking markers, PM severity distributions were observed among the RILs from the Avocet × Francolin#1 and Avocet × Quaiu#3 crosses in the presence or absence of two consistent loci, *QPm.heau-1BL* and *QPm.heau-4BL*. RILs with *QPm.heau-4BL* displayed mean MDS of 38.5% and 35.6% in the Francolin#1 and Quaiu#3 populations, respectively, whereas the mean severities were 53.9 and 53.3% for RILs lacking *QPm.heau-4BL*, respectively (Table 4). In Francolin#1 population, a significant MDS reduction of 12.2–16.3% was observed in RILs with *QPm.heau-4BL*, compared with those without resistance allele across three environments as well as MMDS (Table 4). A significant effect was also found in Quaiu#3 population with a mean PM reduction of 13–21.1% for RILs carrying resistance allele at *QPm.heau-4BL* locus (Table 4). *QPm.heau-1BL* also showed a significant effect on PM at the adult plant stage in both populations. A mean MDS reduction of 16.7–24.6 and 10.7–18.9% was observed in Avocet × Francolin#1 and Avocet × Quaiu#3 RILs with *QPm.heau-1BL*, respectively, compared to RILs without this locus (Table 5).

**Table 4:** *t*-tests for the comparison of mean maximum disease severity in Avocet × Francolin#1 and Avocet × Quaiu#3 RILs when *QPm.heau-4BL* was either absent or present.

	**Avocet × Francolin#1**					**Avocet × Quaiu#3**				
		**Maximum disease severity (Mean)**					**Maximum disease severity (Mean)**			
***QPm.heau-4BL***	**RIL (No.)**	**2014ZZ**	**2015ZZ**	**2015SQ**	**Means**	**RIL (No.)**	**2014SQ**	**2015ZZ**	**2015SQ**	**Means**
Absent	59	63.6 A	51.2 A	45.2 A	53.9 A	74	42.3 A	61.7 A	56.8 A	53.3 A
Present	46	47.3 B	39.0 B	30.5 B	38.5 B	73	29.3 B	40.6 B	37.4 B	35.6 B

*Different letters within each column following the means indicate significant differences based on the t test (P = 0.01); ZZ, Zhengzhou; SQ, Shangqiu*.

**Table 5:** *t*-tests for the comparison of mean maximum disease severity in Avocet × Francolin#1 and Avocet × Quaiu#3 RILs when *QPm.heau-1BL* was either absent or present.

	**Avocet × Francolin#1**					**Avocet × Quaiu#3**				
		**Maximum disease severity (Mean)**					**Maximum disease severity (Mean)**			
***QPm.heau-1BL***	**RIL (No.)**	**2014ZZ**	**2015ZZ**	**2015SQ**	**Means**	**RIL (No.)**	**2014SQ**	**2015ZZ**	**2015SQ**	**Means**
Absent	57	67.7A	52.9A	46.2A	56.2A	67	43.0A	60.3A	57.0A	50.3A
Present	46	43.1B	35.6B	29.5B	36.1B	58	32.3B	41.4B	39.5B	37.6B

*Different letters within each column following the means indicate significant differences based on the t test (P = 0.01); ZZ, Zhengzhou; SQ, Shangqiu*.

## Discussion

Quantitative and qualitative genetic analyses showed that about three to four PM resistance genes conferred partial resistance in Francolin#1 and Quaiu#3 derived RIL populations, respectively. These results corroborated the QTL mapping results. Avocet contributed a consistent QTL in both mapping populations. Most of the QTL identified through bi-parental QTL analyses were also identified by JICIM with the exception of one minor locus on chromosome 1DL from Francolion#1, indicating JICIM was a powerful method for QTL analysis in nested association mapping populations due to an improvement of linkage maps.

The QTL on chromosome 1BL was common in both populations. It was closely linked to the diagnostic marker *csLV46* or *csLV46G22* for known PM APR gene *Pm39*. However, there is only one common marker between the genetic linkage groups on chromosome 1BL for the two populations (Figures 1A,E). Due to the limited number of SSR and DArT markers at present, only three other poorly fit common markers were finally added to the linkage map on 1BL for Avocet × Francolin#1 population, which might lead to the long distance between the markers *csLV46* and *csLV46G22* on the joint linkage map. PM QTL at the same position on 1BL was reported in Massey (Liu et al., 2001) and its derivative USG3209 (Tucker et al., 2007). The gene *Pm39* is known to have pleotropic effects on leaf rust, stripe rust and stem rust (Lillemo et al., 2008; Singh et al., 2013; Kolmer et al., 2015). Previous studies on leaf rust and stripe rust resistance in the two populations have shown that the chromosome 1BL QTL explained 23.0–44.5% and 6.0–43.6% for leaf rust and stripe rust severity variations in Avocet × Francolin#1 and Avocet × Quaiu#3, respectively (Basnet et al., 2014; Lan et al., 2014), indicating that this locus should be the known pleiotropic APR gene *Lr46/Yr29/Pm39* based on its effect on multiple diseases. Moreover, our study shows that the genetic background of the host genotype profoundly influences the expression of *Pm39*; the phenotypic variance attributed to *QPm.heau-1BL* was 20.3–26.1% in Avocet × Francolin#1 population compared to 4.8–11.5% in the Avocet × Quaiu#3 population.

*QPm.heau-1DL*, derived from wheat line Francolion#1, was located on the distal region of chromosome 1DL based on the genetic map of USG3209/Jaypee-1D (http://wheat.pw.usda.gov/cgi-bin/GG3/report.cgi?class=marker;query=∗5721∗; name = *XwPt5721-1D*). The LOD curves indicated that this locus had stable effects on PM resistance across all three environments, and explained 6.1–8.5% of the phenotypic variance. Keller et al. (1999) detected a QTL (*QPm.sfr-1D*) for PM resistance in Swiss winter wheat Forno, flanked by RFLP markers *psr168* and *glk558b*, explaining 8.1–13.7% of phenotypic variances. Bougot et al. (2006) also identified a QTL for PM resistance on chromosome 1D in the French winter wheat line RE9001. It was closely linked with SSR marker *gwm160* with a PVE of 12.6% in a single environment. These QTLs should be different from *QPm.heau-1DL* based on more than 20 cM genetic distance between them (Somers et al., 2004; He et al., 2011; Marone et al., 2013). Thus, it was supposed that *QPm.heau-1DL* was a novel QTL for APR to PM.

*QPm.heau-2DL*, mapped on the distal end of chromosome 2DL, was closely linked to the DArT marker *wPt-667054* and co-located with *Yr54* for stripe rust resistance in Quaiu#3 (Basnet et al., 2014). Keller et al. (1999) detected a QTL, *QPm.sfr-2D*, on chromosome 2DL within the marker interval *psr932-psr331* explaining 8.2–12.2% of phenotypic variances in the Swiss spelt wheat cultivar Oberkulmer. It seems that the QTL from Oberkulmer is located more proximal on chromosome 2DL than *QPm.heau-2DL* (Somers et al., 2004; https://wheat.pw.usda.gov/GG3/). Börner et al. (2002) detected a minor QTL for PM resistance from the synthetic wheat W7984 on 2DL and it was flanked by markers *glk588* and *ksuD23*. This QTL was possibly the same as *QPm.heau-2DL* based on genetic map (http://wheat.pw.usda.gov/GG3/). In addition, two PM QTLs at the distal of chromosome 2DL were also detected to be close to marker *gwm301* in the Swedish winter wheat Folke and German spring wheat Naxos (Lu et al., 2012; Lillemo et al., 2012; Windju et al., 2017). Therefore, known stripe rust gene *Yr54*, which has only a slight effect in seedlings and a moderate effect in adult plants, could be linked to 2DL QTL, or may have pleiotropic effect on powdery mildew resistance. However, fine mapping and/or cloning of *Yr54* will be necessary to resolve it.

A QTL from Avocet was identified in both populations in the current study. This QTL was located between *wPt-6209* and *barc20* on the long arm of chromosome 4B in the same location as QTL for PM resistance in the winter wheat Forno (Keller et al., 1999), synthetic hexaploid wheat W7984 (Börner et al., 2002), Italian wheat Strampelli (Asad et al., 2013) and the Israeli wheat cultivar Oligoculm (Liang et al., 2006) based on an integrated map developed by Marone et al. (2013). The same QTL contributed by Avocet has shown significant effects on stripe rust and leaf rust in the cross Avocet × Pavon in two seasons (1998–1999 and 1999–2000). Although our two populations were also tested for stripe rust and leaf rust in previous studies (Lan et al., 2014; Basnet et al., 2014), the LOD curves at the same location were far from being significant, which is in agreement with finding by Lillemo et al. (2008). Contribution of the 4BL QTL from Avocet explains the RILs showing transgressive segregation for both increased resistance and susceptibility (Figure S1).

Bougot et al. (2006) identified a QTL, *QPm.inra-5BL.2*, in the French wheat cultivar Courtot in the marker interval of *gwm604*-*gwm790*. However, this QTL was more than 40 cM away from *QPm.heau-5BL* based on Somers et al. (2004). Keller et al. (1999) reported a QTL for PM severity on chromosome 5BL in the Swiss spelt cultivar Oberkulmer between *psr580b* and *psr143*. It was ~30 cM from *QPm.inra-5BL.2* (He et al., 2011) and came closer to the terminal end of chromosome, indicating the difference from *QPm.heau-5BL*. Recently, Asad et al. (2013) identified *QPm.caas-5BL.1* and *QPm.caas-5BL.2* in the Italian wheat cultivar Strampelli, flanked by *gwm335-barc331* and *barc331-wmc537*, respectively, which fall within the interval of *QPm.heau-5BL* in the present study. Furthermore, *QPm.heau-5BL* was also located in the region corresponding to *QPm.inra-5BL.1* on chromosome 5B (Bougot et al., 2006; Marone et al., 2013). Lu et al. (2009) detected a significant QTL for stripe rust resistance in a similar chromosomal position corresponding to *QPm.caas-5BL.2*, but *QPm.heau-5BL* did not show significant effect on stripe rust (Lan et al., 2014).

Keller et al. (1999) found a QTL within the AFLP marker interval of *psr167b*-*psr964* on the long arm of chromosome 6B in winter wheat Forno, which should differ from *QPm.heau-6BS* based on the wheat consensus map. Lan et al. (2009) identified a QTL conferring resistance to PM on chromosome 6BS in Chinese winter wheat Bainong 64 on the same position as *QPm.heau-6BS*. The former explained 10.3–13.2% of total phenotypic variance in PM severity in different environments, which is similar to *QPm.heau-6BS* in the present study. In addition, a PM QTL at a similar position on chromosome 6BS was also detected in the Swedish winter wheat Folke (Lillemo et al., 2012).

In this study, two consistent QTL, *QPm.heau-1BL* and *QPm.heau-4BL*, were detected in two populations based on ICIM and JICIM. Furthermore, we were able to identify another locus (*QPm.heau-5BL*) in both populations using JICIM. The detection of *QPm.heau-5BL* is most probably attributed to the higher mapping power of JICIM.

## Conclusion

In this study, we detected six QTL conferring APR to PM across environments, including *QPm.heau-1BL* (*Pm39*), *QPm.heau-1DL, QPm.heau-2DL, QPm.heau-4BL, QPm.heau-5BL*, and *QPm.heau-6BS. QPm.heau-1DL* is likely to be a novel QTL for resistance to PM. Besides *Pm39, QPm.heau-2DL* might be another co-located/pleotropic resistance gene in wheat, which maps to same position as *Yr54*. The closely linked molecular markers for each QTL would be of great significance for QTL pyramiding from the parents Francolin#1 and Quaiu#3 in the future. It may be even better to use RILs that have shown significantly higher level of resistance than the parents.

## Author contributions

YR carried out the experiment and wrote the manuscript. WH, WZ, XC, and DC participated in field trials. CL, BB, and RS performed SSR and DArT genotyping and revised the paper. YR and FC designed the study. All authors have read and approved the final manuscript.

### Conflict of interest statement

The authors declare that the research was conducted in the absence of any commercial or financial relationships that could be construed as a potential conflict of interest.

## Supplementary material

Figure S1 Frequency distribution of Avocet × Francolin#1 **(A)** and Avocet × Quaiu#3 **(B)** recombinant inbred lines (RILs) for powdery mildew maximum disease severities (MDS) in three environments. ZZ14, Zhengzhou 2014; ZZ15, Zhengzhou 2015; SQ14, Shangqiu 2014; SQ15, Shangqiu 2015.

Table S1 Analysis of variance for maximum disease severity values for RILs generated from Avocet × Francolin#1 and Avocet × Quaiu#3 crosses.

Table S2 Estimated number of resistance genes conferring adult plant resistance to powdery mildew in 196 Avocet × Francolin#1 and 195 Avocet × Quaiu#3 F_5_recombinant inbred lines (RILs) based on Mendelian segregation analysis.

Table S3 Range and mean maximum disease severities for parents and Avocet × Francolin#1 and Avocet × Quaiu#3 F_5_ recombinant inbred lines (RILs), and narrow-sense heritability estimates during 2014–2015 and 2015–2016 cropping seasons at Zhengzhou and Shangqiu, as well as the estimated minimum number of segregating resistance genes using the Wright method.

Table S4 summary of the main features of the wheat linkage maps in Avocet × Francolin#1 and Avocet × Quaiu#3 populations.
