# Supplementary figures and images for: QTL Analysis and Nested Association Mapping for Adult Plant Resistance to Powdery Mildew in Two Bread Wheat Populations
Source: Front Plant Sci. 2017 Jul 27;8:1212. doi: 10.3389/fpls.2017.01212 (PMC5529384; doi:10.3389/fpls.2017.01212)

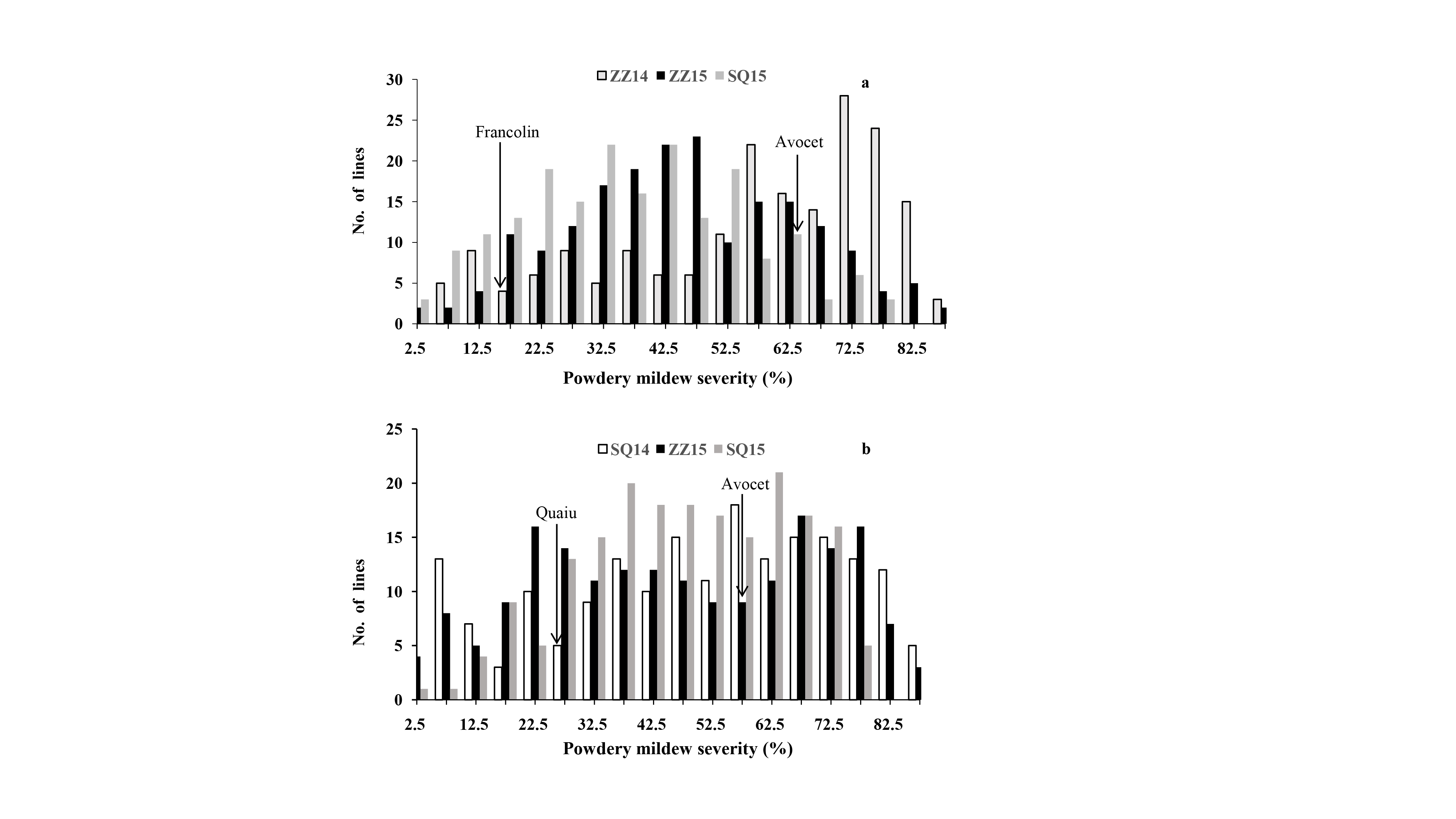

Supplement: Figure S1 — Frequency distribution of Avocet × Francolin#1 (A) and Avocet × Quaiu#3 (B) recombinant inbred lines (RILs) for powdery mildew maximum disease severities (MDS) in three environments. ZZ14, Zhengzhou 2014; ZZ15, Zhengzhou 2015; SQ14, Shangqiu 2014; SQ15, Shangqiu 2015. [file Image1.JPEG]
