# Supplementary material for: QTL Analysis and Nested Association Mapping for Adult Plant Resistance to Powdery Mildew in Two Bread Wheat Populations
Source: Front Plant Sci. 2017 Jul 27;8:1212. doi: 10.3389/fpls.2017.01212 (PMC5529384; doi:10.3389/fpls.2017.01212)
Supplement: Table S1 — Analysis of variance for maximum disease severity values for RILs generated from Avocet × Francolin#1 and Avocet × Quaiu#3 crosses. [file Table1.DOC]

Table S1 Analysis of variance for maximum disease severity values for RILs generated from Avocet × Francolin#1 and Avocet × Quaiu#3 crosses

| Population | Source of variation | *df* | Mean square | *F* value | *P* value |
| --- | --- | --- | --- | --- | --- |
| Avocet /Francolin#1 | Lines | 195 | 2868.99 | 12.5** | < 0.0001 |
|  | Environments | 2 | 47395.57 | 207.1** | < 0.0001 |
|  | Replicates (Environment) | 6 | 3854.81 | 16.8** | < 0.0001 |
|  | Lines × environments | 384 | 348.71 | 1.5** | < 0.0001 |
|  | Error | 1188 | 228.87 |  |  |
| Avocet/Quaiu#3 | Lines | 194 | 3417.8 | 13.1** | < 0.0001 |
|  | Environments | 2 | 34520.6 | 132.4** | < 0.0001 |
|  | Replicates (Environment) | 6 | 3108.5 | 11.9** | < 0.0001 |
|  | Lines × environments | 376 | 392.2 | 1.5** | < 0.0001 |
|  | Error | 1139 |  |  |  |
