# Supplementary material for: QTL Analysis and Nested Association Mapping for Adult Plant Resistance to Powdery Mildew in Two Bread Wheat Populations
Source: Front Plant Sci. 2017 Jul 27;8:1212. doi: 10.3389/fpls.2017.01212 (PMC5529384; doi:10.3389/fpls.2017.01212)
Supplement: Table S2 — Estimated number of resistance genes conferring adult plant resistance to powdery mildew in 196 Avocet × Francolin#1 and 195 Avocet × Quaiu#3 F5recombinant inbred lines (RILs) based on Mendelian segregation analysis. [file Table2.DOC]

Table S2 Estimated number of resistance genes conferring adult plant resistance to powdery mildew in 196 Avocet × Francolin#1 and 195 Avocet × Quaiu#3 F_5_ recombinant inbred lines (RILs) based on Mendelian segregation analysis

|  |  | No. of F_5_ RILs | | | *P* value ^a^ | | | |
| --- | --- | --- | --- | --- | --- | --- | --- | --- |
| Population | Location | Resistant | Susceptible-intermediate | Missing | 2 genes | 3 genes | 4 genes | 5 genes |
| Avocet /Francolin#1 | Zhengzhou 2014 | 25 | 171 | 0 | <0.01 | 0.26 | <0.01 | <0.01 |
|  | Zhengzhou 2015 | 7 | 186 | 3 | <0.01 | <0.01 | 0.45 | 0.22 |
|  | Shangqiu 2015 | 9 | 184 | 3 | <0.01 | <0.01 | 0.93 | 0.03 |
| Avocet /Quaiu#3 | Shangqiu 2014 | 33 | 162 | 0 | 0.09 | <0.01 | <0.01 | <0.01 |
|  | Zhengzhou 2015 | 27 | 162 | 6 | 0.01 | 0.07 | <0.01 | <0.01 |
|  | Shangqiu 2015 | 27 | 162 | 6 | 0.01 | 0.07 | <0.01 | <0.01 |

^a^ *P* value is for *χ^2^* test. The expected radio of RILs grouped under resistant and susceptible-intermediate are 0.22:0.78, 0.103:0.897, 0.048:0.952, and 0.023:0.977, respectively, for 2, 3, 4, and 5 independent genes in the F_5_ generation.
