# Supplementary material for: QTL Analysis and Nested Association Mapping for Adult Plant Resistance to Powdery Mildew in Two Bread Wheat Populations
Source: Front Plant Sci. 2017 Jul 27;8:1212. doi: 10.3389/fpls.2017.01212 (PMC5529384; doi:10.3389/fpls.2017.01212)
Supplement: Table S3 — Range and mean maximum disease severities for parents and Avocet × Francolin#1 and Avocet × Quaiu#3 F5 recombinant inbred lines (RILs), and narrow-sense heritability estimates during 2014–2015 and 2015–2016 cropping seasons at Zhengzhou and Shangqiu, as well as the estimated minimum number of segregating resistance genes using the Wright method. [file Table3.DOC]

Table S3 Range and mean maximum disease severities for parents and Avocet × Francolin#1 and Avocet × Quaiu#3 F_5_ recombinant inbred lines (RILs), and narrow-sense heritability estimates during 2014-2015 and 2015-2016 cropping seasons at Zhengzhou and Shangqiu, as well as the estimated minimum number of segregating resistance genes using the Wright method.

| Population | Parents/parameter | Zhengzhou 2014 | Zhengzhou 2015 | Shangqiu2014 | Shangqiu 2015 |
| --- | --- | --- | --- | --- | --- |
| Avocet /Francolin#1 | Francolin#1 | 22.7 | 14 | - | 9.8 |
|  | Avocet | 78.3 | 67.5 | - | 48.3 |
|  | Population mean | 56 | 46.4 | - | 38 |
|  | Low range | 3.7 | 3 | - | 5 |
|  | High range | 90 | 88.3 | - | 80 |
|  | *h^2^* | 0.84 | 0.81 | - | 0.75 |
|  | No. of genes | 2.7 | 4.0 | - | 3.1 |
| Avocet /Quaiu#3 | Quaiu#3 | - | 31.7 | 26.8 | 30 |
|  | Avocet | - | 53.3 | 48.3 | 75 |
|  | Population mean | - | 51.2 | 36.4 | 47.5 |
|  | Low range | - | 5 | 3.7 | 3.7 |
|  | High range | - | 90 | 76.7 | 88.3 |
|  | *h^2^* | - | 0.9 | 0.64 | 0.85 |
|  | No. of genes | - | 2.9 | 2.3 | 2.6 |
