# Supplementary material for: QTL Analysis and Nested Association Mapping for Adult Plant Resistance to Powdery Mildew in Two Bread Wheat Populations
Source: Front Plant Sci. 2017 Jul 27;8:1212. doi: 10.3389/fpls.2017.01212 (PMC5529384; doi:10.3389/fpls.2017.01212)
Supplement: Table S4 — summary of the main features of the wheat linkage maps in Avocet × Francolin#1 and Avocet × Quaiu#3 populations. [file Table4.DOC]

Table S4 summary of the main features of the wheat linkage maps in Avocet × Francolin#1 and Avocet × Quaiu#3 populations

|  |  | Avocet × Francolin#1 |  |  |  | Avocet × Quaiu#3 |  |
| --- | --- | --- | --- | --- | --- | --- | --- |
| Chromosome | No. of markers | Length (cM) | Distance (cM/marker) |  | No. of markers | Length (cM) | Distance (cM/marker) |
| 1A | 42 | 120.9 | 2.9 |  | 33 | 130.9 | 4.0 |
| 2A | 26 | 156.6 | 6.0 |  | 19 | 100.5 | 5.3 |
| 3A | 16 | 90.4 | 5.6 |  | 9 | 122.7 | 13.6 |
| 4A | 21 | 82.9 | 3.9 |  | 35 | 224.2 | 6.4 |
| 5A | 19 | 112.1 | 5.9 |  | 16 | 111.2 | 6.9 |
| 6A | 47 | 92.6 | 2.0 |  | 29 | 59.4 | 2.0 |
| 7A | 25 | 123.3 | 4.9 |  | 26 | 180.7 | 7.0 |
| Genome A | 196 | 778.6 | 4.0 |  | 167 | 929.5 | 5.6 |
| 1B | 39 | 157.6 | 4.0 |  | 20 | 131.2 | 6.6 |
| 2B | 50 | 380.1 | 7.6 |  | 38 | 269.2 | 7.1 |
| 3B | 62 | 322.9 | 5.2 |  | 23 | 157.8 | 6.9 |
| 4B | 24 | 144.0 | 6.0 |  | 11 | 32.7 | 3.0 |
| 5B | 41 | 150.5 | 3.7 |  | 24 | 201.7 | 8.4 |
| 6B | 43 | 191.6 | 4.5 |  | 34 | 132.6 | 3.9 |
| 7B | 19 | 95.6 | 5.0 |  | 40 | 170.5 | 4.3 |
| Genome B | 278 | 1442.4 | 5.2 |  | 190 | 1095.8 | 5.8 |
| 1D | 29 | 94.4 | 3.3 |  | 8 | 54.9 | 6.9 |
| 2D | 16 | 114.0 | 7.1 |  | 31 | 147.7 | 4.8 |
| 3D | 23 | 58.2 | 2.5 |  | 18 | 164.5 | 9.1 |
| 4D | 9 | 50.6 | 5.6 |  | 3 | 27.3 | 9.1 |
| 5D | 3 | 10.2 | 3.4 |  | 9 | 93.8 | 10.4 |
| 6D | 4 | 25.6 | 6.4 |  | 8 | 88.1 | 11.0 |
| 7D | 34 | 97.1 | 2.9 |  | 5 | 26.6 | 5.3 |
| Genome B | 118 | 450.1 | 3.8 |  | 82 | 603.0 | 7.4 |
| Genomes A, B, D | 592 | 2671.1 | 4.5 |  | 439 | 2628.3 | 6.0 |
